# Individual factors and perceived community characteristics in relation to mental health and mental well-being

**DOI:** 10.1186/s12889-015-2590-8

**Published:** 2015-12-12

**Authors:** Helen McAneney, Mark A. Tully, Ruth F. Hunter, Anne Kouvonen, Philip Veal, Michael Stevenson, Frank Kee

**Affiliations:** Centre for Public Health, School of Medicine, Dentistry and Biomedical Sciences, Queen’s University Belfast, Belfast, Northern Ireland, UK; UKCRC Centre of Excellence for Public Health (NI), Queen’s University Belfast, Belfast, Northern Ireland, UK; Department of Social Research, University of Helsinki, Helsinki, Finland; University of Social Sciences and Humanities, Faculty in Wroclaw, Wroclaw, Poland; Public Health Agency, Northern Ireland, UK

**Keywords:** Mental well-being, Mental health, Neighbourhood deprivation, Individual social capital, Social epidemiology, Self-rated health, United Kingdom

## Abstract

**Background:**

It has been argued that though correlated with mental health, mental well-being is a distinct entity. Despite the wealth of literature on mental health, less is known about mental well-being. Mental health is something experienced by individuals, whereas mental well-being can be assessed at the population level. Accordingly it is important to differentiate the individual and population level factors (environmental and social) that could be associated with mental health and well-being, and as people living in deprived areas have a higher prevalence of poor mental health, these relationships should be compared across different levels of neighbourhood deprivation.

**Methods:**

A cross-sectional representative random sample of 1,209 adults from 62 Super Output Areas (SOAs) in Belfast, Northern Ireland (Feb 2010 – Jan 2011) were recruited in the PARC Study. Interview-administered questionnaires recorded data on socio-demographic characteristics, health-related behaviours, individual social capital, self-rated health, mental health (SF-8) and mental well-being (WEMWBS). Multi-variable linear regression analyses, with inclusion of clustering by SOAs, were used to explore the associations between individual and perceived community characteristics and mental health and mental well-being, and to investigate how these associations differed by the level of neighbourhood deprivation.

**Results:**

Thirty-eight and 30 % of variability in the measures of mental well-being and mental health, respectively, could be explained by individual factors and the perceived community characteristics. In the total sample and stratified by neighbourhood deprivation, age, marital status and self-rated health were associated with both mental health and well-being, with the ‘social connections’ and local area satisfaction elements of social capital also emerging as explanatory variables. An increase of +1 in EQ-5D-3 L was associated with +1SD of the population mean in both mental health and well-being. Similarly, a change from ‘very dissatisfied’ to ‘very satisfied’ for local area satisfaction would result in +8.75 for mental well-being, but only in the more affluent of areas.

**Conclusions:**

Self-rated health was associated with both mental health and mental well-being. Of the individual social capital explanatory variables, ‘social connections’ was more important for mental well-being. Although similarities in the explanatory variables of mental health and mental well-being exist, socio-ecological interventions designed to improve them may not have equivalent impacts in rich and poor neighbourhoods.

## Background

The World Health Organization defines mental health as “a state of well-being in which every individual realizes his or her own potential, can cope with the normal stresses of life, can work productively and fruitfully, and is able to make a contribution to her or his community” [1]. A related concept, mental well-being, has been receiving increasing attention in recent years, is moving up the policy agenda [2, 3], and is becoming increasingly recognised as a key public health indicator [4]. There is no agreed definition of mental well-being, but it is generally seen as covering both the subjective experience of affect and life satisfaction, as well as psychological functioning, good relationships with others and self-realisation [5, 6]. Mental health and mental well-being can be seen to form two distinct, but correlated, continua in populations [7–9]. Mental well-being (vs. mental illness) can be seen as the positive component of mental health [10]. However an absence of mental illness in an individual may not necessarily represent positive mental well-being, and vice versa, individuals with mental illness may also have positive mental well-being [11, 12]. Huppert has argued that the independence of mental illness and mental well-being suggests that their determinants may differ [13]. It is plausible that different individual and community level factors could affect mental health and mental well-being in different ways. Accordingly it is important to differentiate the individual and perceived social and environmental variables associated with both.

Features of the neighbourhood social environment, such as safety/violence, social connections or cohesion, and informal social norms have been associated with mental health outcomes, particularly depression [14, 15]. In a similar way, individual perceptions of neighbourhood social processes have been associated with depressive symptoms [16]. Furthermore, low individual social capital, which can be seen to refer to cognitive (support, reciprocity, sharing, and trust), and structural (the extent and intensity of associational links) aspects related to social relationships of individuals [17, 18], has been associated with common mental disorders [19]. Living in a deprived or socioeconomically disadvantaged neighbourhood is also associated with poor mental health, although the effect sizes have varied across studies and have generally been modest [14]. Emerging evidence also suggests that resilience of a locality, that is high levels of social capital, may help to explain why one poor neighbourhood has lower mortality than other equally deprived areas [20]. Some studies indicate that neighbourhood deprivation may affect mental health over and above individual-level socio-economic factors [21] and Jokela [22] has considered the causal relationship of neighbourhood deprivation and poorer health. Less is known, however, about mental well-being, Mason and Kearnes [23] explored mental well-being and physical activity in deprived neighbourhoods in Glasgow, UK and found the greatest potential benefits to mental well-being were for those from deprived neighbourhoods. These findings build further upon those of Bond et al. [6] who found that for people living in deprived areas, the perception of the housing and neighbourhoods were associated with mental well-being. Therefore the level of neighbourhood deprivation could also moderate the relationship between perceived social neighbourhood factors and mental health and well-being.

Most previous studies assessing the effects of neighbourhood social environment on mental health or mental well-being have used measures of mental illness (e.g., depression) rather than positive aspects of mental well-being [14]. In a recent review on social capital and mental well-being in older adults, all included studies showed positive association between social capital and some aspects of mental well-being, but the definitions and instruments to measure mental well-being varied widely [10]. This review further showed that there are no studies investigating the relationship between individual social capital and measures specifically designed to assess mental well-being such as the Warwick-Edinburgh Mental Well-Being Scale (WEMWBS) [10]. However, in a recent study, people’s residential psychosocial environments were associated with higher mental well-being measured by WEMWBS [6].

### Aim of the study

The aim of this study is to examine the extent to which individual factors and perceived neighbourhood social environment are associated with mental health and mental well-being, and to compare these relationships across different levels of neighbourhood deprivation.

We use a representative sample of a population living in a specific municipality, including information on its neighbourhoods. Unlike previous studies, we compare the explanatory variables associated with mental health and mental well-being, using measures specifically designed to assess mental health (SF8 MCS) and mental well-being (WEMWBS).

## Methods

### Study sample

We used data from the Physical Activity and the Rejuvenation of Connswater (PARC) study baseline survey. The PARC study is a natural experiment evaluation of the Connswater Community Greenway (CCG) [24]. The CCG is a £32 million investment in East Belfast, Northern Ireland, UK, which aims to regenerate the local environment by creating a 9 km linear park. One aim of the CCG is to physically reconnect communities, in order to improve the health and well-being of the approximately 40,000 residents living nearby. In summary, a representative stratified random sample of 1,209 adults completed a household interview (Feb 2010 – Jan 2011). Households where randomly selected through the Postcode Address File and a resident (aged over 16 year) was randomly selected for interview. The study was approved by the Office for Research Ethics, Northern Ireland (09/NIR02/66).

### Outcomes

A range of self-reported outcome measures were collected during the interview-administered survey.

#### Socio-demographic characteristics

Individual socio-demographic variables included age, gender, household income, marital status (married, co-habiting, single, divorced, widowed), housing tenure (owner occupied, rented, other), educational attainment (whether educated to degree/diploma level or not), and employment status (unemployed, employed, retired, student, other). Access to a bicycle and the number of vehicles available for use by the household were also recorded.

#### Self-rated health

Self-rated health was measured using the EQ-5D-3 L instrument. Data were used to generate an EQ-5D score using 5 dimensions (mobility, self-care, usual activities, pain/discomfort, and anxiety/depression) each with three response categories to indicate no, some or extreme problems to create a summary score and an EQ-VAS, which assessed the participants’ health state on the day of interview on a visual analogue scale ranging from 0 to 100, with higher scores indicating the self-rating of a better health state on the day of interview [25, 26].

#### Health-related behaviours

Self-reported smoking status (never, ex- or current smoker) and frequency of alcohol intake were recorded. Physical activity was assessed using the Global Physical Activity Questionnaire (GPAQ) developed by the World Health Organisation, with categories of low, moderate and high physical activity derived by the standardised scoring protocol [27].

#### (Individual) social capital

Social capital, as reflected in civic engagement, neighbourliness, social networks and support, and perceptions of the local area, was assessed using the instrument employed in the UK General Household Survey [28, 29]. Thus social capital measures comprised 16 items: one 5-point Likert item assessing local area satisfaction, a scale of eight 5-point Likert items assessing views of problems within local area, one scored on a 4-point ordinal scale for ‘trust’/neighbourliness, one dichotomous item on ‘civic participation’, one dichotomous item on ‘social participation’ and a Likert scale on for ‘social networks and social support’ scored on four 5-point Likert items.

#### Neighbourhood deprivation

Participants’ postcode of current address was linked to the census Super Output Areas (SOAs [30] http://www.nisra.gov.uk/deprivation/super_output_areas.htm) and thence to scores on the 2010 Northern Ireland Multiple Deprivation Measure (NIMDM) [31]. The NIMDM provides an area-level measure of deprivation using a weighted combination of seven domains: income, employment, health, education, proximity, living environment and crime and disorder [31]. The higher the NIMDM score the more deprived the area. SOAs are ranked according to the NIMDM score and placed into quintiles from most (1) to least (5) deprived.

#### Mental health and mental well-being

Mental health was assessed using the validated Short-Form 8 Health Survey (SF-8), comprising 8 items from which the mental composite score (MCS) was created [32]. For SF-8 a single item is used to measure each of the eight domains of health in the SF-36 and SF-12 health surveys instruments (physical functioning, role physical, bodily pain, general health, vitality, social functioning, role emotional and mental health). The summary measure for the mental health was created following the guidelines led out within the SF-8 manual, that is, (i) applying the appropriate SF-36 V2 scale score to each of the SF-8 item response categories, (ii) weighting the SF-8 scales using the appropriate regression coefficient (from the general U.S. population) and (iii) aggregating the scores and adding the regression intercept. Thus the SF-8 MCS are standardised to the SF-36 MCS in the general U.S. population with mean of 50 (SD 10) [32].

Mental well-being was assessed using the recently developed Warwick Edinburgh Mental Well-Being Scale [5, 33]. This scale comprises 14 positively worded statements with five response categories scored from one (never) to five (all of the time). Following WEMWBS guidelines, scores were summed to give a total between 14 (poorest mental well-being) and 70 (best mental well-being), with higher scores indicating greater well-being. The scale is treated as a continuous, quasi-normally distributed variable. It covers most aspects of positive mental health (positive thoughts and feelings) currently in the literature, including both hedonic and eudaimonic dimensions. It has been validated on a representative general population sample for use within the UK [5, 34].

### Statistical analysis

The associations between individual and perceived neighbourhood attributes (independent variables), and how these differed across neighbourhood deprivation (NIMDM) strata, and the mental health (SF-8 MCS) and mental well-being (WEMWBS) dependent variables were assess using multi-variable linear regression. We initially verified the assumptions for multi-variable linear regression (data independence, being normally and identically distributed). A range of potential confounding variables were considered for inclusion in our multi-variable models, including (i) socio-demographic characteristics [6, 11, 20, 35]: gender, age, marital status, housing tenure, educational attainment, employment status, bicycle and vehicle availability and income; (ii) health status [6, 18]: EQ-5D-3 L and EQ-VAS; (iii) health behaviours including smoking status, frequency of alcohol intake, and physical activity [23, 36–38]. To address the stated aim, social capital variables were included within the model, and the sample stratified by neighbourhood deprivation (NIMDM), which have been explored in various aspects as factors for both mental health and mental well-being, see for instance Bond et al. [6], Verhaeghe et al. [18], De Silva et al. [19] and Crump et al. [21].

To explore what to include in an explanatory set of variables, multi-variable linear regression models were initially assessed (separately for mental health and mental well-being) using the stepwise, backward and forward methods, with inclusion/exclusion of explanatory variables based on the F-statistic. By considering the total and stratified samples, categorical covariates (marital status, housing tenure, employment status, smoking status and highest level of education) where dichotomised according to the dominant category and the covariates retained in these separate sub-models were collated to form the core explanatory set of variables. To address the stated aim, all individual social capital variables where retained in the model. A check for parallelism across strata was undertaken for each covariate. Modification effects across neighbourhood deprivation levels were examined by investigation of the interaction of individual independent variables and the neighbourhood deprivation variable for both dependent variables. Lastly, using this core explanatory set, a multi-variable linear regression model, with inclusion of potential area level clustering by SOAs, was derived for the total and stratified samples for the mental health and the mental well-being dependent variables. Analyses were undertaken using SPSS version 19 and STATA version 13.

## Results

### Descriptive statistics

The characteristics of the final study sample are presented in Table 1. Within East Belfast, comparatively affluent neighbourhoods are adjacent to neighbourhoods where people experience material deprivation. The sample is from a total of 62 SOAs, with 10, 13, 5, 10 and 24 SOAs being within the NIMDM quintiles from most to least deprived respectively. The mean number of participants per SOA was 19.5 (SD 7.7).

**Table 1:** Characteristics of the study sample (*n* = 1209 PARC Study, East Belfast, UK; Feb 2010 – Jan 2011)

Characteristic	n	Percent	Mean	St. Dev.
Gender				
Male	490	40.5		
Female	719	59.5		
Age, years ^a^	1192	98.6	50.36	19.94
Employment Status				
Unemployed	60	5.0		
Employed/Retired/Student/Other	1149	95.0		
Marital Status ^a^				
Married/cohabiting	604	50.0		
Single/Divorced/Widowed	603	49.9		
Education to Degree/Diploma Level ^a^				
Yes	396	32.8		
No	811	67.1		
Housing Tenure ^a^				
Owner Occupied	845	69.9		
Rent/Other	353	29.2		
Smoking ^a^				
Ever	594	49.1		
Never	613	50.7		
Alcohol intake (frequency) ^a^				
Weekly or more	583	48.2		
Monthly	266	22.0		
Yearly	58	4.8		
Not in last year	289	23.9		
PA category ^a^				
Low level	462	38.2		
Medium level	373	30.9		
High Level	372	30.8		
Number of vehicles access to				
0	301	24.9		
1	555	45.9		
2 +	353	29.2		
Access to bicycle ^a^				
Yes	402	33.3		
No	806	66.7		
NIMDM ^b^				
1 – most deprived	217	17.9		
2	223	18.4		
3	94	7.8		
4	232	19.2		
5 – least deprived	443	36.6		

^a^ Data not complete for all cases (*n* < 1209)

^b^ NIMDM: Northern Ireland Multiple Deprivation Measure [31]

Descriptive statistics for the two dependent variables are presented in Tables 2 and 3. From the sample of 1,209 participants, SF-8 MCS data were available for 1,201 participants and WEMWBS for 1,203 participants. The dependent variables were initially compared to population/normative scores and an unpaired *t*-test conducted to compare population versus sample means (p-value two tailed). The WEMWBS were comparable with available normative data for the UK population, both as a total sample and by gender and age [33]. In contrast, the SF-8 scores from the sample were generally lower than those of other UK populations [39].

**Table 2:** Descriptive statistics of SF-8 MCS within the PARC Study sample versus normative health scores

Variable	N	Sample Mean ^a^ (95 % CI)	Min-Max	Population Mean ^b,c^	*t*-test unpaired *p*-value (two tailed)
Total	1201	**48.31** (47.74, 48.88)	13–65	49.2	**0.001**
Gender					
Male	486	**49.35** (48.46, 50.24)	17–65	50.6	**0.003**
Female	715	47.61 (46.87, 48.35)	13–65	48.0	0.358
Age category					
16–24	87	**49.77** (47.63, 51.91)	18–61	46.4	**0.004**
25–34	214	**48.62** (47.23, 50.01)	13–65	47.4	**0.000**
35–44	214	47.88 (46.56, 49.20)	19–65	49.1	0.079
45–54	197	**47.48** (46.02, 48.94)	15–62	49.2	**0.019**
55–64	161	**46.62** (44.92, 48.32)	18–63	51.1	**0.000**
65–74	165	**49.15** (47.70, 50.60)	21–64	52.3	**0.000**
75 plus	163	**49.54** (48.16, 50.92)	26–65	51.9	**0.005**
NIMDM 2010					
1 -most deprived	215	45.61 (44.02, 47.20)	13–65	-	-
2	221	47.33 (45.94, 48.72)	15–65	-	-
3	94	46.84 (44.77, 48.90)	17–62	-	-
4	232	48.73 (47.47, 49.99)	15–63	-	-
5 - least deprived	439	50.23 (49.42, 51.03)	19–65	-	-

*SF-8 MCS* Short-Form 8 Health Survey Mental Composite Score, *PARC* Physical Activity and the Rejuvenation of Connswater, *NIMDM* Northern Ireland Multiple Deprivation Measure [31]

^a^ Bold sample mean values denote statistically significant (*p* < 0.05) difference to the population mean by the unpaired *t*-test (two-tailed)

^b^ Population norms taken from Ware et al. [39]

^c^ Age group of 18–24 years in population data

**Table 3:** Descriptive statistics of the WEMWBS within the PARC Study sample versus normative health scores

Variable	N	Sample Mean ^a^ (95 % CI)	Min-Max	Population Mean ^b^	*t*-test unpaired *p*-value (two tailed)
Total	1203	50.84 (50.35-51.33)	15–70	50.7	0.664
Gender					
Male	486	51.11 (50.32-51.90)	18–70	51.3	0.731
Female	717	50.65 (50.02-51.28)	15–70	50.3	0.438
Age category					
16–24	87	52.15 (50.60-53.70)	28–68	51.7	0.644
25–34	214	**52.36** (51.30-53.42)	29–70	50.1	**0.003**
35–44	215	50.51 (49.29-51.73)	15–70	49.7	0.289
45–54	197	49.36 (48.09-50.63)	21–70	49.5	0.873
55–64	162	**49.25** (47.82-50.68)	18–70	51.4	**0.016**
65–74	166	51.98 (50.74-53.22)	30–69	52.4	0.631
75 +	162	50.78 (49.42-52.14)	19–70	51.2	0.754
NIMDM 2010					
1 - most deprived	215	48.86 (47.51-50.21)	15–70	-	-
2	223	50.68 (49.46-51.90)	21–70	-	-
3	94	48.53 (46.91-50.15)	24–69	-	-
4	232	50.52 (49.45-51.59)	19–70	-	-
5 - least deprived	439	52.56 (51.84-53.28)	27–70	-	-

*WEMWBS* Warwick-Edinburgh Mental Well-Being Scale, *PARC* Physical Activity and the Rejuvenation of Connswater, *NIMDM* Northern Ireland Multiple Deprivation Measure [31]

^a^ Bold sample mean values denote a statistically significant (*p*-value <0.05) difference to the population mean by the unpaired *t*-test (two-tailed)

^b^ Population norms taken from Stewart-Brown & Janmohamed [33]

### Individual factors and perceived community characteristics associated with mental health and mental well-being

Results of the final multi-variable linear regression on the SF-8 MCS and WEMWBS dependent variables, clustered by SOAs, are summarised in Tables 4 and 5 respectively, for both the total sample and stratified by neighbourhood deprivation (NIMDM). Detailed for each explanatory variable are the regression coefficients (B), 95 % confidence intervals and their statistical significance in the final model. The last column in each table contains the statistical significance of the interaction of individual independent variables and neighbourhood deprivation strata on the dependent, showing there is a variation in the effect of some variables according to the level of neighbourhood deprivation.

**Table 4:** Summary of the linear regression with clustering by SOA on SF-8 MCS by NIMDM strata

Stratified sample	Total		NIMDM 1		NIMDM 2		NIMDM 3		NIMDM 4		NIMDM 5		Interaction
Independent variable	B^a^ (95 % CI)	p^b^	B^a^ (95 % CI)	p^b^	B^a^ (95 % CI)	p^b^	B^a^ (95 % CI)	p^b^	B^a^ (95 % CI)	p^b^	B^a^ (95 % CI)	p^b^	*p* value (Indep var * NIMDM)
Constant	21.72 (15.28, 28.16)	***	14.92 (−3.35, 33.19)		12.05 (−4.24, 28.35)		35.15 (8.40, 61.89)	*	16.18 (−0.32, 32.67)		31.67 (26.21, 48.08)	***	
*Socio-demographic*													
Gender	−0.79 (−2.00, 0.43)		−0.27 (−4.02, 3.48)		−0.85 (−3.83, 2.14)		−2.48 (−9.69, 4.74)		−1.94 (−5.52, 1.63)		−1.12 (−2.75, 0.43)		
Age	0.11 (0.08, 0.15)	***	0.11 (0.04, 0.19)	**	0.13 (0.03, 0.22)	*	0.12 (0.04, 0.19)	*	0.08 (0.02, 0.15)	*	0.14 (0.06, 0.16)	***	
Married/Cohabiting	1.208 (0.09, 2.07)	*	−1.07 (−4.04, 1.90)		1.63 (−0.88, 4.14)		−2.38 (−9.60, 4.83)		2.47 (−0.01, 4.95)		1.27 (−1.09, 2.27)		
Accommodation-Own	−0.28 (−1.59, 1.02)		−0.66 (−3.37, 2.05)		−0.25 (−3.16, 2.65)		0.62 (−2.36, 3.60)		0.59 (−0.99, 2.17)		−1.08 (−2.94, 2.87)		
Education to Degree Level	−0.28 (−1.54, 0.97)		4.12 (0.93, 7.31)	*	2.07 (−4.56, 0.41)		−2.74 (−5.04, −0.43)	*	1.60 (−1.35, 4.54)		−1.45 (−2.59, 0.60)		**
Unemployed	−2.38 (−5.79, 1.04)		−3.10 (−11.38, 5.12)		−2.20 (−8.74, 4.34)		7.44 (−4.91, 19.79)		2.11 (−6.68, 10.90)		−6.22 (−9.29, 1.81)		
Bicycle access	0.46 (−0.81, 1.73)		1.78 (−3.14, 6.71)		0.25 (−4.22, 4.71)		−0.60 (−5.14, 3.95)		1.96 (−0.56, 4.49)		−0.27 (−0.02, 0.02)		
No. Vehicles	0.72 (−0.06, 1.49)		0.77 (−1.9,3.15)		−0.21 (−2.32, 1.91)		0.12 (−3.51, 3.76)		0.82 (−0.25, 1.90)		0.88 (−0.32, 2.01)		
Income	0.08 (−0.27, 0.43)		−0.12 (−1.62, 1.38)		0.94 (0.32, 1.56)	**	0.85 (−0.53, 2.24)		−0.31 (−1.06, 0.45)		0.14 (−0.47, 0.75)		**
*Self-Rated Health*													
EQ-VAS	0.16 (0.12, 0.21)	***	0.25 (0.11, 0.38)	**	0.22 (0.15, 0.29)	***	0.12 (−0.08, 0.32)		0.14 (0.06, 0.21)	**	0.10 (0.04, 0.15)	*	***
EQ-5D-3 L	13.09 (10.04, 16.14)	***	13.39 (7.77, 19.02)	***	8.27 (−0.47, 17.01)		7.74 (−3.57, 19.04)		14.56 (9.29, 19.83)	***	13.27 (8.44, 16.88)	***	
*Health Related Behaviours*													
Never smoked	−0.44 (−1.51, 0.63)		−0.38 (−4.66, 3.91)		−1.33 (−3.42, 0.75)		−2.76 (−9.02, 3.50)		1.33 (−1.07, 3.74)		−0.75 (−1.78, 1.51)		
Alcohol	0.03 (−0.26, 0.33)		−0.06 (−1.08, 0.96)		0.71 (−0.07, 1.49)		−0.52 (−2.32, 1.29)		−0.30 (−0.81, 0.22)		−0.02 (−0.39, 0.44)		
*Social Capital*													
Local Area Satisfaction	−0.75 (−1.48, −0.02)	*	−0.68 (−2.64, 1.28)		0.10 (−1.21, 1.40)		−2.62 (−6.78, 1.53)		−0.66 (−1.78, 0.46)		−1.40 (−2.79, −0.09)		
Local Area Problems	−0.05 (−0.17, 0.07)		−0.14 (−0.41, 0.12)		0.13 (−0.06, 0.32)		−0.01 (−0.57, 0.54)		0.10 (−0.31, 0.51)		−0.12 (−0.42, 0.08)		
‘Social connections’	−0.08 (−0.31, 0.16)		0.28 (−0.14, 0.70)		−0.22 (−0.67, 0.24)		0.40 (−1.20, 1.99)		0.01 (−0.49, 0.50)		−0.34 (−0.69, −0.01)		
Trust	−0.07 (−0.77, 0.63)		0.34 (−2.05, 2.72)		−0.64 (−1.86, 0.58)		−1.36 (−5.65, 2.94)		1.28 (0.03, 2.53)	*	−0.62 (−1.57, 0.38)		
Civic Participation	0.53 (−0.64, 1.70)		0.00 (−4.13, 4.13)		−0.53 (−3.68, 2.61)		−1.43 (−5.16, 2.30)		0.41 (−2.05, 2.88)		1.45 (−0.78, 2.41)		
Social Participation	0.15 (−0.76, 1.06)		−0.57 (−3.64, 2.50)		1.14 (−1.17, 3.45)		−2.79 (−6.57, 0.99)		0.17 (−2.64, 2.99)		0.58 (−0.92, 2.25)		

*PARC*: Physical Activity and the Rejuvenation of Connswater

*EQ-VAS*: Visual Analogue Scale of self-rated health; EQ-5D-3 L: 5 dimension with 3 levels of response [25, 26]

*NIMDM*: Northern Ireland Multiple Deprivation Measure [31]

^a^ Regression coefficients, ^b^ Significance level: * *p* < 0.05, ** *p* < 0.01, *** *p* < 0.001

Coding of dependent variables

SF8- MCS: continuous variable with increase in score implying better mental health

Coding of independent variables

Gender: 1 = male, 2 = female

Age: in years

Married/Cohabiting: 0 = false, 1 = true

Accommodation own: 1 = owner occupied, 2 = other/rent

Education to Degree Level: 0 = false, 1 = true

Unemployed: 0 = false, 1 = true

Bicycle access: 0 false, 1 = true

No. Vehicles: numeric

Income: 9 categories of increasing amount

EQ-VAS: 0 to 100 (higher values imply better health)

EQ-5D-3 L: continuous variable (higher values imply better health)

Never Smoked: 0 = false, 1 = true

Alcohol: ranked categories of frequency from 1 = almost every day to 8 = not at all in the last 12 months

Local Area Satisfaction: scale from 1 = very satisfied to 5 = very dissatisfied

Local Area Problems: summative of 8 Llikert items to a Likert scale 5 – 40 (higher values better)

‘Social connections’: summative of 4 Likert items to a Likert scale of 4 – 20 (lower values better)

Trust: 4 point ordinal scale, 1 = very likely to 4 = not at all likely

Civic Participation: 0 = no, 1 = yes

Social Participation: 0 = no, 1 = yes

Health Related Behaviours

**Table 5:** Summary of the linear regression with clustering by SOA on WEMWBS by NIMDM strata

Stratified sample	Total		NIMDM 1		NIMDM 2		NIMDM 3		NIMDM 4		NIMDM 5		Interaction
Independent variable	B^a^ (95 % CI)	p^b^	B^a^ (95 % CI)	p^b^	B^a^ (95 % CI)	p^b^	B^a^ (95 % CI)	p^b^	B^a^ (95 % CI)	p^b^	B^a^ (95 % CI)	p^b^	*p* value (Indep var * NIMDM)
Constant	40.08 (34.64, 45.51)	***	41.77 (25.68, 57.87)	***	35.39 (23.68, 47.10)	***	36.22 (14.94, 57.51)	**	42.89 (23.50, 62.27)	***	38.62 (28.26, 48.97)	***	
*Socio-demographic*													
Gender	−0.14 (−1.13,0.85)		−0.93 (−4.55, 2.69)		−1.40 (−4.01, 1.22)		0.56 (−4.57, 5.68)		−0.35 (−2.30, 1.61)		−0.09 (−1.84, 1.08)		
Age	0.05 (0.01, 0.08)	**	0.13 (0.03, 0.24)	*	0.06 (−0.03, 0.16)		−0.04 (−0.17, 0.09)		0.01 (−0.09, 0.09)		0.05 (−0.01, 0.08)		
Married/ Cohabiting	1.40 (0.28, 2.52)	*	1.58 (−2.12, 5.27)		1.49 (−0.69, 3.67)		3.27 (−4.33, 10.86)		3.09 (−0.82, 6.99)		0.47 (−1.48, 1.61)		
Accommodation-Own	−1.01 (−2.23, 0.21)		−1.05 (−3.82, 1.73)		−1.65 (−4.49, 1.20)		−1.37 (−3.79 1.06)		−1.81 (−4.36, 0.74)		0.26 (−3.25, 2.09)		
Education to Degree Level	1.48 (0.45, 2.51)	**	3.32 (−0.93, 7.58)		0.83 (−2.79, 4.45)		3.13 (−1.98, 8.24)		1.51 (0.13, 2.90)	*	1.29 (−0.33, 2.61)		
Unemployed	−3.68 (−5.81, −1.55)	***	−3.64 (−8.30, 1.02)		−4.90 (−8.81, −0.99)	*	−1.54 (−9.14, 6.06)		1.35 (−6.44, 9.14)		−6.43 (−9.53, 0.66)	**	
Bicycle access	−0.60 (−1.75, 0.55)		−1.41 (−4.27, 1.45)		−1.74 (−6.44, 2.96)		−1.03 (−7.99, 5.93)		1.23 (−0.89, 3.35)		0.08 (−0.02, 0.01)		**
No. Vehicles	−0.03 (−0.62, 0.56)		1.03 (−1.09, 3.15)		0.06 (−1.69, 1.81)		−1.45 (−4.14, 1.23)		−0.76 (−1.81, 0.30)		0.14 (−0.72, 1.42)		
Income	−0.04 (−0.32, 0.23)		−0.24 (−1.27, 0.79)		0.24 (−0.40, 0.87)		−0.39 (−1.39, 0.61)		−0.20 (−0.94, 0.53)		0.05 (-0.42, 0.52)		
*Self-Rated Health*													
EQ-VAS	0.14 (0.10, 0.17)	***	0.18 (0.08, 0.27)	**	0.16 (0.09, 0.22)	***	0.17 (−0.08, 0.42)		0.10 (0.02, 0.17)	*	0.10 (0.06, 0.15)	***	*
EQ-5D-3 L	6.19 (3.34, 9.03)	***	4.41 (0.23, 8.59)	*	5.95 (−1.68, 13.58)		7.90 (−4.25, 20.06)		8.67 (0.04, 17.31)	*	4.22 (−1.23, 6.53)		
*Health Related Behaviours*													
Never smoked	056 (−0.64, 1.75)		0.36 (−3.16, 3.87)		−1.86 (−4.69, 0.97)		0.31 (−2.46, 3.08)		1.82 (−1.00, 4.63)		1.64 (0.09, 3.11)		
Alcohol	0.20 (−0.03, 0.43)		0.07 (−0.64, 0.78)		0.46 (−0.14,1.07)		−0.24 (−0.90, 0.42)		0.11 (−0.45, 0.68)		0.06 (−0.33, 0.44)		
*Social Capital*													
Local Area Satisfaction	−1.06 (−1.65, −0.47)	***	−0.73 (−2.81,1.06)		−0.78 (−2.01, 0.44)		−0.96 (−4.75, 2.84)		−1.75 (−2.95, −0.56)	**	−1.39 (−3.06, −0.57)	*	
Local Area Problems	−0.07 (−0.22, 0.08)		−0.24 (−0.64, 0.16)		0.13 (−0.23, 0.49)		0.18 (−0.49, 0.86)		−0.08 (−0.58, 0.42)		0.10 (−0.16, 0.30)		
‘Social connections’	−0.37 (−0.60, −0.14)	**	−0.42 (−1.09, 0.25)		−0.4958 (−1.02, −0.04)		−0.28 (−1.37, 0.82)		−0.10 (−0.48, 0.28)		−0.52 (−0.94, −0.32)	*	
Trust	−0.12 (−0.70, 0.45)		−0.17 (−1.32, 0.99)		0.17 (−0.95, 1.30)		−0.11 (−3.31, 3.09)		−0.04 (−1.95, 1.87)		−0.09 (−1.15, 0.65)		
Civic Participation	−0.53 (−1.49, 0.43)		−2.50 (−4.98, −0.03)	*	−1.59 (−4.61, 1.43)		−2.94 (−7.14, 0.26)		−1.19 (−3.51, 1.13)		0.60 (−0.83, 2.10)		**
Social Participation	−0.92 (−1.85, 0.02)		−0.72 (−3.69, 2.25)		−0.88 (−3.29, 1.52)		−2.52 (−6.61, 1.56)		−2.08 (−5.12, 0.95)		−0.12 (−1.89, 1.02)		

PARC: Physical Activity and the Rejuvenation of Connswater

EQ-VAS: Visual Analogue Scale of self-rated health; EQ-5D-3 L: 5 dimension with 3 levels of response [25, 26]

NIMDM: Northern Ireland Multiple Deprivation Measure [31]

^a^ Regression coefficients, ^b^ Significance level: * *p* < 0.05, ** *p* < 0.01, *** *p* < 0.001

Coding of dependent variables

WEMWBS: 14 – 70 with increase in score implying better mental well-being

Coding of independent variables

Gender: 1 = male, 2 = female

Age: in years

Married/Cohabiting: 0 = false, 1 = true

Accommodation own: 1 = owner occupied, 2 = other/rent

Education to Degree Level: 0 = false, 1 = true

Unemployed: 0 = false, 1 = true

Bicycle access: 0 false, 1 = true

No. Vehicles: numeric

Income: 9 categories of increasing amount

EQ-VAS: 0 to 100 (higher values imply better health)

EQ-5D-3 L: continuous variable (higher values imply better health)

Never Smoked: 0 = false, 1 = true

Alcohol: ranked categories of frequency from 1 = almost every day to 8 = not at all in the last 12 months

Local Area Satisfaction: scale from 1 = very satisfied to 5 = very dissatisfied

Local Area Problems: summative of 8 Llikert items to a Likert scale 5 – 40 (higher values better)

‘Social connections’: summative of 4 Likert items to a Likert scale of 4 – 20 (lower values better)

Trust: 4 point ordinal scale, 1 = very likely to 4 = not at all likely

Civic Participation: 0 = no, 1 = yes

Social Participation: 0 = no, 1 = yes

#### Total sample

The adjusted coefficient of multiple determination (R^2^) indicates that 38 % and 30 % of variability in the mental health and mental well-being, respectively, could be explained by the individual factors and the perceived community characteristics. Age, marital status, self-rated health and the local area satisfaction element of social capital were significant explanatory variables of both mental health and well-being. Thus being older, living with a partner, in good self-perceived health and having a higher satisfaction of the local area were associated with better mental health and mental well-being. In addition, obtaining education to degree level, not being unemployed and having better social connection were significantly associated with better mental well-being.

#### NIMDM strata

Across neighbourhood deprivation strata (most to least deprived), 50 %, 47 %, 51 %, 44 % & 27 % and 37 %, 40 %, 54 %, 35 %, & 21 % of variability in the mental health and well-being respectively, could be accounted for by the final model. For both mental health and well-being, the final model accounted for a greater percentage of variability of the dependent variables in the strata from the more deprived neighbourhoods than for the total sample.

Age remains a significant explanatory variable in all strata, such that being older is associated with better mental health regardless of area level deprivation, and self-rated health remains significant in nearly all strata. Education to degree level emerges as a positive and significant explanatory variable of mental health in the most deprived areas, but the coefficient changes sign in the middle NIMDM3 category, that is, being educated to degree level predicts a lower level of mental health for those in the middle quintile (neither extremely deprived nor affluent). The trust element of social capital emerges as a significant explanatory variable for those from the more affluent areas but with a negative relationship, that is, lower levels of trust was associated with better mental health. Income was a positive explanatory variable of mental health but only in the mid-tier of area deprivation, NIMDM 3. Thus for those from areas in the middle category of deprivation, being older, having higher household income and better self-rated health were associated with better mental health.

For mental well-being, the significant explanatory variables from the total sample are also retained as statistically significant explanatory variables across strata, with the exception of marital status. The social capital aspects of local area satisfaction or social connections are significant explanatory variables but only in the more affluent of the quintiles of neighbourhood deprivation. Being involved in civic participation is associated negatively with mental well-being in the most deprived of areas.

Overall, when the statistically significant explanatory variables of mental health and well-being are compared per stratum, there are commonalities, but also certain variations. For instance, for those from the most deprived areas, age and self-rated health are common explanatory variables of both dependent variables, but civic participation is associated negatively with mental well-being and mental health positively with education to degree level. For those from the most affluent areas, self-rated health is common explanatory variable for both dependent variables. On the other hand, age is more important for mental health and unemployment and the social capital elements of local area satisfaction and social connections are more important for mental well-being.

## Discussion

In 2011 Giles articulated the ‘top ten’ research questions which should be priorities for social science. Among the most important of these was the question, how does the social become biological (and vice versa) [40]. This reflects the ethos behind the “No health without mental health” UK cross-government strategy for people of all ages [3]. The temptation is to think only of physical health, but to do so would ignore the importance of mental health and mental well-being which, though often less visible, are themselves strong explanatory variables of physical health and longevity [41].

Research literature, has for the main part been concerned with mental (ill) health and although there is widespread agreement that mental health is more than the absence of clinically defined mental illness, there is still ongoing debate as to the elements that constitute positive mental health or mental well-being. The WEMWBS considers both the hedonic and eudemonic dimensions of well-being, and has been validated for use in the United Kingdom [5]. Although a population level measure and not designed as a clinical tool, it does appear to be sensitive to changes in mental well-being at the individual level and allows for the investigation of the determinants of mental well-being [http://www2.warwick.ac.uk/fac/med/research/platform/wemwbs/].

The current study aimed to examine the extent to which individual factors and perceived neighbourhood social environment were associated with mental health and well-being, and compared these relationships across different levels of neighbourhood deprivation. The current study made use of measures specifically designed to assess mental health (SF8 MCS) and mental well-being (WEMWBS), making use of a representative sample of a population living in a specific municipality, including information on its neighbourhoods.

We found that a range of individual and perceived community characteristics accounted for a reasonably high proportion of variability in mental health and mental well-being (38 % and 30 % respectively), but the association power of these covariates differed among people living in deprived and more affluent neighbourhoods. For instance, for a 20 years increase in age, a participant would achieve a 1 point significant increase in their mental well-being score, whereas this increase was over two and a half fold greater at 2.6 points when participants from the most deprived strata were considered, but not a significant explanatory variable in any other of the deprivation strata. However, age was a significant explanatory variable of better mental health in both the total and stratified sample, of comparable magnitude regardless of the level of neighbourhood deprivation (1.6 to 2.2 point increase in mental health per 20 year increase in the age of the participant). In contrast, not being unemployed had a 3.7 point increase in mental well-being for the general sample, but when considering the neighbourhood deprivation strata, this increased almost two fold to 6.43 points for participants from the most affluent of areas. This improvement in mental well-being is almost one standard deviation of the population mean, which some have deemed as an adequate definition for the difference between low, average and high mental well-being [6]. Whereas for mental health, being educated to degree level only had a significant association for those living in the most deprived of areas, at the second largest magnitude of over 4 points, only improved upon further by an increase of one level in self-rated health EQ-5D-3 L or 17 % within the EQ-VAS. These differences may be important to those designing interventions to improve mental well-being if what works most effectively for one group or area is not what works for others, and it may be necessary to take account of such differences to avoid widening social inequalities. Our findings are in agreement with the conclusions of the 2009 WHO report on mental health, resilience and inequalities, “A greater understanding of inequalities is also crucial to recognizing the limits of what promoting positive mental health can achieve. Positive mental health does confer considerable protection and advantage, but it does so predominantly among those with equal levels of resources.” [20].

Some explanatory variables, such as self-rated health, are common to both mental health and mental well-being, were better perceived health is associated positively with both mental health and well-being, though an elevation to a higher level on the EQ-5D-3 L scale produced an increase of 6.19 points on the mental well-being scale (i.e., 0.71 of a SD, again almost a change from low to average or average to high mental well-being), but an increase of 13.09 points (greater than one (1.17) standard deviation of the population mean) would result for mental health. Others, including indicators of individual social capital, such as satisfaction with the local area and ‘social connections’, are more important for mental well-being than mental health when considered across neighbourhood deprivation strata. That is, greater local area satisfaction was found to be significant explanatory variable for better mental well-being for the total sample (+1.06 per category increase in local area satisfaction) and those from the more affluent neighbourhoods (+1.75 for NIMDM4, and +1.39 for NIMDM5); in contrast, it was found to be a significant indicator of better mental health only for the total sample (+0.75per increase in local area satisfaction category). In other words, if a participant from an area more affluent than average levels of neighbourhood deprivation (NIMDM4) where to alter their perception of their local area satisfaction from ‘very dissatisfied’ to‘ very satisfied’, the participant’s mental well-being score would increase by more than one standard deviation of the population average (5x1.75 = 8.75), sufficient to convert low or average mental well-being to average or high, if mental well-being were to be trichotomised as in Bond et al. [6]. Although social capital is a placed-based ecological concept, most directly conceived as related to one’s neighbourhood or to places where individuals interact socially [42], our data corroborate other studies that demonstrate its importance to individual health outcomes. But what we have yet to empirically discern is whether regeneration programmes aimed at neighbourhoods can shift average population mental well-being. If they do, it would still be important to know whether the number of people with mental illness would be changed and how such effects might be mediated.

Previous research has shown that mental health problems are more common in areas of material deprivation [15, 21]. By the same token poor mental well-being is consistently associated with unemployment, low education, low income and low material standard of living [11]. Although there is growing evidence on the role of social capital in improving mental well-being through promoting community cohesion, social interaction and social trust [43], of these covariates, in our study only social connections (through social networks and social support) and local area satisfaction were associated with mental well-being in fully adjusted models. Our findings are in accord with those of Gidlow et al., for whom other social capital indicators of trust and social participation did not emerge as explanatory variables of mental health or mental well-being [35]. In contrast, Propper et al. found that it was the characteristics of individuals and individual households that were more important (than place itself) in determining levels of common mental disorders [44].

Two recent studies have explored that relationship between housing, neighbourhoods and mental well-being (using WEMWBS) for residents of 15 deprived areas within Glasgow [6, 45]. The first found that perceived (poor) aesthetic quality of the neighbourhood was associated with lower well-being, while satisfaction with the local area was strongly associated with higher mental well-being [6]. Indeed, the perceived internal reputation of the neighbourhood, but not the external reputation was positively associated with mental well-being [45]. Our own results concerning satisfaction with the local area aspects of social capital accord with these findings. In contrast, Gale et al. found no association between area-level deprivation and mental well-being, assessed using the WEMWBS [46], but their study only examined the relationship in 69–78 year olds.

Mental health is not just the absence of mental illness [4, 20], while mental well-being, though a related concept, is a measure of the hedonic (positive feelings or positive affect such as subjective wellbeing, life satisfaction, happiness) and eudemonic (positive functioning such as engagement, fulfilment, sense of meaning, social wellbeing) dimensions [5, 20, 33]. As mental health and well-being move up the policy agenda as it has both social and health benefits:

“Mental health promotion does have a role in preventing mental health problems, notably anxiety, depression, drug and alcohol dependence and suicide. But mental health promotion also has a wider range of health and social benefits. These include improved physical health, increased emotional resilience, greater social inclusion and participation and higher productivity.” [4, pg28]

mechanisms by which to promote mental health through developing theoretical and empirically sound complex interventions are beginning to appear in the literature [47]. Nonetheless, as summarised in the 2009 WHO report ‘Mental health, resilience and inequalities’,

“While there is much that can be done to improve mental health, doing so will depend less on specific interventions, valuable as these may be, and more on a policy sea change, in which policy makers across all sectors think in terms of ‘mental health impact’.” [20, pg 4],

so it is a challenge to everyone, not just those involved with interventions, to make an impact on mental health.

### Strengths and limitations of study

Strengths of this study include a representative sample, the sampling of adjacent areas from one city, from affluent to deprived neighbourhoods assessed using a reliable area-level measure of deprivation, and sampling taking place over a single year, limiting any potential seasonal affects on mental health or well-being. As the academic community moves away from the measurement of the presence/absence of mental (ill) health, toward that of mental well-being, this paper is well placed to guide individuals forward and deliver pathways of thought, as the two concepts have been compared using measures specifically designed to access mental health (SF8 MCS) and mental well-being (WEMWBS), in parallel in a specific municipality.

Perhaps a limitation of our study is that more sophisticated causal analyses have not been attempted using, for example, structural equation models that might better distinguish mediators from moderators, however this would be more appropriate for longitudinal data. Further limitations, such as the lack of area-level measurement of social capital and the cross-sectional nature of the study must also be acknowledged, and with this the difficulty to establish what is cause and what is effect. It is possible that some behavioural variables, such as physical activity (e.g., walking in the neighbourhood), are more likely in neighbourhoods with high social capital but that in turn such behaviours themselves give more opportunities for building social capital. This demonstrates the complex nature that associations may have were even reasons to be engaged (or not) in physical activity is a complex matter and one which is difficult to unpick [48]. Our purpose on the other hand was to explore the extent to which variation in measures of mental health and mental well-being and their explanatory variables differed in subgroups of the population.

## Conclusions

By sampling a population across a range of neighbourhood deprivation levels in one city, and accounting for clustering within local areas, we have been able to compare the explanatory variables of both mental health and mental well-being. Although certain personal attributes, such as better self-perceived health, being of older age, and marital status of married or cohabiting were all associated with better health and well-being, when the total sample was considered, educated to degree level and not being unemployed where only associated with better mental well-being. Individual social capital explanatory variables of ‘local area satisfaction’ and ‘social connections’ emerge as explanatory variables of mental well-being across the total sample of all strata but were only associated with mental health in the affluent areas when considered by strata of neighbourhood deprivation. Although there are similarities in the explanatory variables of mental health and mental well-being, socio-ecological interventions designed to improve them may not have equivalent impacts in rich and poor neighbourhoods.

## Abbreviations

CCG: Connswater Community Greenway
GPAQ: Global Physical Activity Questionnaire
NIMDM: Northern Ireland Multiple Deprivation Measure
PARC: Physical Activity and the Rejuvenation of Connswater
SF-8 MCS: Short-Form 8 Health Survey Mental Composite Score
SOAs: Super Output Areas
WEMWBS: Warwick-Edinburgh Mental Well-Being Scale
